# Pileup-hi: an ultra-high-throughput, customizable alignment pileup program for large datasets

**DOI:** 10.1093/bioinformatics/btag721

**Published:** 2026-09-28

**Authors:** E Piliper, A L Greninger

**Affiliations:** Department of Laboratory Medicine and Pathology, University of Washington Medical Center, Seattle, WA 98109, United States; Department of Laboratory Medicine and Pathology, University of Washington Medical Center, Seattle, WA 98109, United States; Vaccine and Infectious Disease Division, Fred Hutchinson Cancer Research Center, Seattle, WA 98109, United States

## Abstract

**Motivation:**

Recent advancements in next-generation sequencing have combined massive data throughput with high read accuracy to facilitate large-scale, rapid, and sensitive analysis of genetic variation. High-depth, high-quality platforms such as the Illumina NovaSeq X and Ultima Genomics UG 100 are increasingly used in time-sensitive clinical contexts such as cancer screening to detect mutations as low as 0.01% without sequence error correction. This process usually involves the bioinformatic construction of a pileup, or a list of nucleotides aligned to one or more positions in a sequence alignment, to identify variants. Efficient pileup software is required to process large-scale sequencing datasets rapidly to allow for timely clinical decision-making. While foundational to many analytical pipelines, the de facto standard pileup software *samtools mpileup* faces scalability challenges with larger datasets and is restricted to one output format.

**Results:**

We present *pileup-hi*, a multi-threaded pileup engine that is scalable to alignments containing billions of reads and extendable to support custom output formats. When set to emit the default mpileup format, *pileup-hi* is up to 13× faster than samtools mpileup, 3.2× faster than sambamba mpileup, and up to 8× faster than *perbase base-depth*. The default output of *pileup-hi* has binary equivalence to the output of *samtools mpileup* across benchmark files and a variety of samtools regression tests. We present a new pileup-derived format that provides depth-invariant data storage proportional only to the reference genome length and number of unique indels.

**Availability and implementation:**

*Pileup-hi* is implemented in the Rust programming language and distributed as open-source code and precompiled binaries. Source code and installation instructions can be found at https://github.com/greninger-lab/pileup-hi.

## 1 Introduction

Novel next-generation sequencers with large, cost-effective throughput and high read accuracy are increasingly being adopted in clinical and exploratory genomics. Ultima Genomics UG 100 platform offers $1/Gb sequencing cost and has also shown scalability in ultra-deep sequencing in liquid biopsy (Almogy *et al.* 2022, Cheng *et al.* 2022, Polonis *et al.* 2025). PacBio’s Revio system stands as an accurate long-read sequencing solution and has been used to resolve chromosomal rearrangements and other structural variants in human genetics (Eisfeldt *et al.* 2024). Additionally, Illumina’s NovaSeq X can yield up to 52 billion reads per flow cell (Illumina 2026), has been shown to generate ∼94% bases with ≥ Q30 (Höjer *et al.* 2025), and is advertised to deliver 85% of bases with ≥ Q40 with the newer XLEAP-SBS chemistry (Illumina 2024).

These sequencing workflows require computational methods to identify sequence variations relative to a reference genome, typically necessitating alignment and storage in the ubiquitous Sequence Alignment/Map (SAM) format and its binary counterpart, BAM (Li *et al.* 2009). The standard approach for extracting alignment-aware query-reference differences from these formats is the pileup algorithm, which aggregates query nucleotides and quality scores at specific reference positions.

Implementations of the pileup algorithm, such as the ones found in the *samtools/bcftools “*mpileup” commands, are foundational to many bioinformatics pipelines and utilities (Kim *et al.* 2018, Poplin *et al.* 2018a, 2018b, Picard toolkit 2019, nf-core/viralrecon n.d.) and remain the standard for retrieving aligned nucleotide information from SAM/BAM formats (Li *et al.* 2009, Danecek *et al.* 2021). However, as datasets scale, some limitations have emerged. Specifically, the algorithm’s typical restriction to a single thread for disk read, data processing, and disk write greatly lengthens runtime and forces the sequential processing of potentially billions of reference coordinates. Furthermore, because the core pileup algorithm responsible for resolving CIGAR strings is coupled to a singular, verbose output format that lists every query nucleotide, output sizes scale with alignment depth. Alternative, less verbose output formats could still meet end-user needs while drastically reducing disk I/O and storage overhead.

*perbase*, a multi-threaded software package built upon *samtools’ htslib*, has emerged to generate broad alignment metrics for alignments by transforming pileup data (Stadick 2026): Although demonstrating scalability and output modularity improvements to the core pileup algorithm, it is not directly applicable to variant calling. Alternatively, while it is possible to run *samtools mpileup* on multiple genomic intervals in parallel via shell scripting, it is not natively offered by samtools, requires custom code, and is not implemented in any package validated against *samtools mpileup* output.

Other software packages in this space carry their own limitations: *Sambamba mpileup* (Tarasov *et al.* 2015), a parallel wrapper around *samtools mpileup* implemented in the D programming language, has been advertised to speed up pileup generation but has not been demonstrated to yield identical output and has since been marked “not recommended” by its maintainers. *Bam-readcount* (Khanna *et al.* 2022), a C++ wrapper around *samtools mpileup* oriented towards variant detection, is only single-threaded.

To address these limitations, we present *pileup-hi*, a high-throughput pileup library and command-line interface program implemented in Rust. *pileup-hi* utilizes a data-oriented design and native multi-threading to reduce processing time of both high-depth and large-reference alignments while maintaining a minimal memory footprint. *pileup-hi* can be installed as a binary program ready to process SAM/BAM files or incorporated into a pre-existing Rust project as a library.

## 2 Implementation and algorithms

The pileup algorithm iterates over every coordinate of a given alignment reference/region, recording the query nucleotides present at each position (Fig. 1A). This process involves the management of a buffer that holds reads with alignments overlapping the current coordinate position and discards those processed during previous iterations. While existing solutions implement this buffer as a linked list for removal of reads in O(1) time, *pileup-hi* instead uses a dynamically-resizing array that performs read removal in batches, reducing the frequency of memory allocations and granting the higher cache-locality of contiguous memory. Batch removal is triggered when 77% of a buffer’s reads are stale, meaning they overlap positions already processed in previous iterations. *pileup-hi*, like *samtools mpileup*, also supports R1/R2 overlap correction, alignment-aware quality adjustment via base alignment quality (BAQ), as well as limiting the size of the buffer to a given depth to reduce memory usage. Depth limitation functions the same as in *samtools mpileup*, where a maximum number of reads are sampled per position: reads encountered once the buffer is full are discarded from analysis.

**Figure 1:**
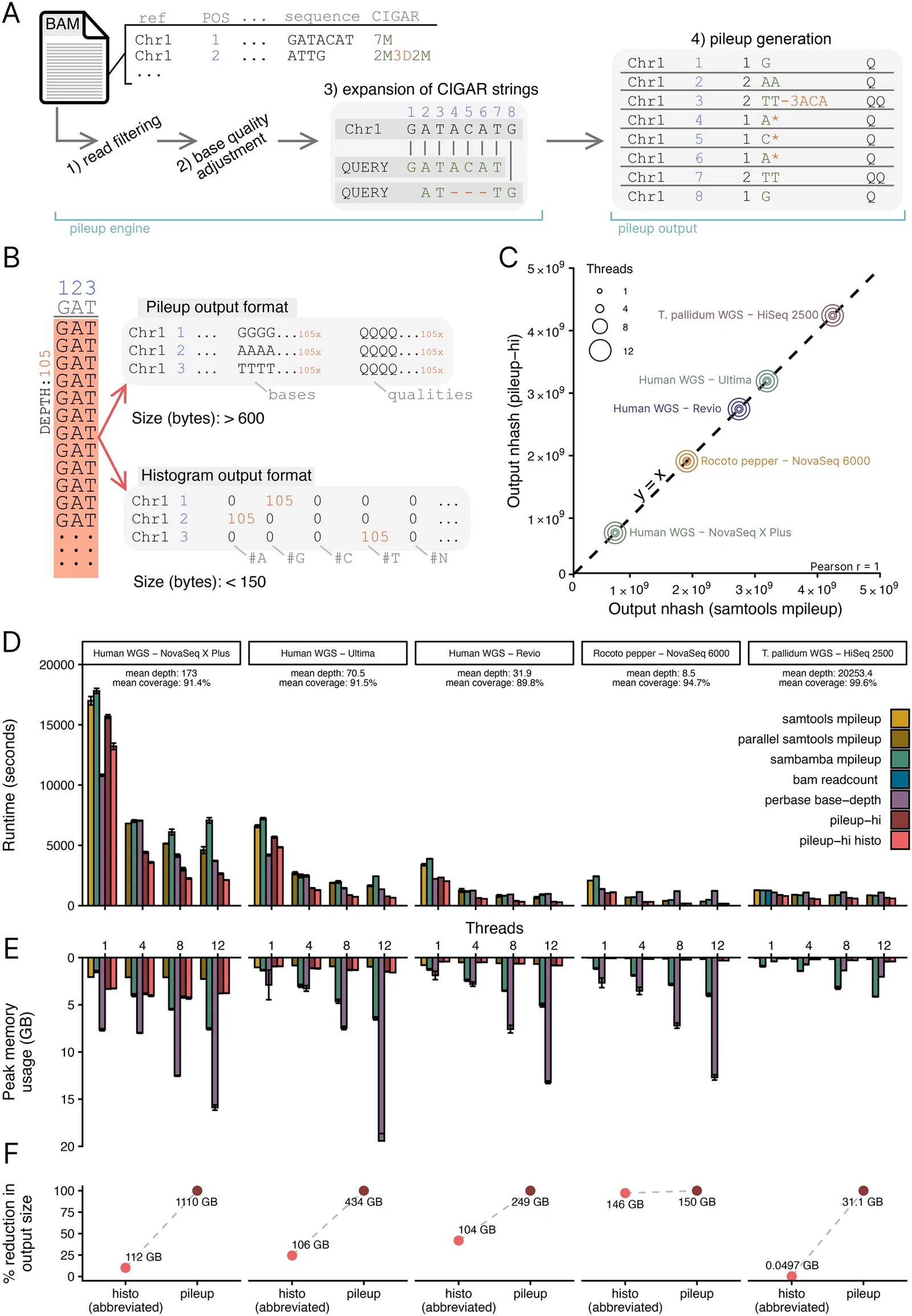
Overview of pileup-hi and comparison against existing software. (A) Overview of the pileup generation process. The pileup engine receives an input BAM, which is then filtered for queries passing user-specified criteria and optionally quality-adjusted. Every base of each remaining query is mapped to individual reference positions by considering the query’s CIGAR string. With this information, the core pileup output format is constructed: Each position has a list of bases, insertions, and deletions. Capital ‘Q” characters represent Phred score values. (B) Overview of new ‘histo” output format relative to pileup format. The orange box displays query bases aligned to the 3-base reference. Formats are shown with some columns removed (ellipses) for brevity. (C) Comparison of output file hashes between *samtools mpileup* and *pileup-hi*. Output files generated by *pileup-hi* and *samtools mpileup* were hashed by the BLAKE3 algorithm and encoded into integer only-values (nhashes) in R for plotting. Circle centers represent nhash values. (D) Total runtime per file in seconds comparing *pileup-hi*, *samtools mpileup* (single and multi-threaded), and *perbase base-depth*. (E) Peak memory usage in gigabytes per file. (F) Percent difference in final output file size between the standard pileup (abbreviated as “plp”) and ‘histo” formats. Measurements for runtime and memory usage were recorded in triplicate. Error bars show standard error.

*pileup-hi* can process an alignment in single-threaded or multi-threaded mode, with the latter requiring indexed alignments. The single-threaded mode is similar to *samtools mpileup* in that one thread is responsible for reading and processing alignments, as well as output. In multi-threaded mode, the reference is split into discrete genomic chunks assigned to independent threads. To ensure accurate R1/R2 overlap correction across boundaries, threads processing mid-reference chunks will perform a ‘look-back’ by rewinding up to twice the maximum read length to capture upstream mates. Each thread writes its results to a temporary file, which are then merged upon completion. This approach allows for synchronization and consolidation of per-thread outputs without the bottleneck imposed by a concurrent queue.

*pileup-hi* is written in Rust and is installable as a command-line program. The architecture of *pileup-hi* decouples core BAM reading/buffer management machinery from the code responsible for output generation: an interface is provided for developers to create custom output formats that can be generated directly from *pileup-hi’*s in-memory data, skipping expensive and unnecessary parsing of the default pileup output. *pileup-hi* includes one such alternative format, ‘histo’, that returns a *count* of bases rather than the pileup’s *list*, which can save significant space for deep sequencing datasets (Fig. 1B). *pileup-hi’*s source code can be found at https://github.com/greninger-lab/pileup-hi.

## 3 Results

We benchmarked the performance of *pileup-hi*, *samtools mpileup*, and *perbase base-depth* across five datasets. We also benchmarked a parallelized implementation of samtools mpileup via a custom shell script (“parallel samtools mpileup”), since this approach has been a standard practice in the bioinformatics community for more than a decade. Test datasets included: one Illumina NovaSeq X Plus WGS run on human HepG2 cells (DRR793869, mean depth = 173.0, Ishida *et al.* 2026), one Ultima UG100 WGS run on a human B-lymphocyte cell line from NIST (SRR36374445, mean depth = 70.5, Santuari *et al.* 2025), one PacBio Revio WGS run on normal human duodenal tissue by the National Institutes of Standards and Technology (NIST, SRR30646149, mean depth = 31.9, (McDaniel *et al.* 2025), one Illumina NovaSeq 6000 WGS run on *Capsicum pubescens* (rocoto pepper, SRR19895870, mean depth = 8.5, Liu *et al.* 2023), and one Illumina HiSeq 2500 WGS of *Treponema pallidum* (ERR2756169, mean depth = 20 253, Beale *et al.* 2019). Additional information on these datasets can be found in Table S1, available as supplementary data at *Bioinformatics* online. Software versions and dataset preparation can be found in the Supplementary Methods, available as supplementary data at *Bioinformatics* online.

### 3.1 Pileup-hi reduces runtime against perbase and single- and multi-threaded samtools mpileup

We measured peak memory usage and total runtime on all datasets across all threetools in triplicate (Fig. 1D-E, Table S2, available as supplementary data at *Bioinformatics* online). To ensure fairness, performance comparisons mentioned in this text are relative to *pileup-hi* outputting the standard mpileup format. To ensure that throughput comparisons were not arbitrarily bottlenecked by disk speed, output of benchmarked tools was discarded for performance testing. *pileup-hi* was faster than *samtools mpileup* by up to 12.6 × (Rocoto pepper, 12 threads vs one thread), and faster than *per-base base-depth* (run with the same thread count) by up to 7.4 × (Rocoto pepper, both using 12 threads). *pileup-hi* was also faster than *sambamba mpileup* by up to 3.2 × (Human WGS—Ultima, both using 12 threads). Compared to the parallelized *samtools mpileup* script at equivalent thread counts, *pileup-hi* still reduced runtime by 1.8–2.1×. *Bam-readcount* exceeded the 8-hour runtime cutoff for all samples except *T. pallidum* WGS, where it was 1.4× slower than single-threaded *pileup-hi.*

*pileup-hi* never exceeded 5 GB of memory usage across all thread counts and input files tested (mean = 1.04 GB), while *perbase base-depth* and *sambamba mpileup* used up to 20 GB of memory (mean = 6.66) and 7.65 GB of memory (mean = 3.28), respectively. Maximum fold-difference in peak memory usage between *pileup-hi* and *perbase base-depth* for a given file was 42×. While we kept *perbase* memory settings at their defaults for fairness, it should be noted that adjusting -c to 50 000 (default 10 00 000) reduced peak memory usage from 20 GB to only 19.9 (mean = 4.96, Table S2, available as supplementary data at *Bioinformatics* online). *samtools mpileup* consumed the least memory across all files and threadcounts, followed by *pileup-hi* at a maximum delta of 1.73 GB.

### 3.2 Pileup-hi yields identical output to samtools mpileup

To verify accurate reproduction of the mpileup algorithm, we compared the outputs of *pileup-hi* and *samtools mpileup* using the BLAKE3 (*b3sum*) hashing algorithm (BLAKE3 team [2019] 2026). The resulting hashes were identical across all datasets, including instances where *pileup-hi* utilized multiple worker threads, demonstrating binary equivalence across hundreds of gigabytes of output data (Fig. 1C, Table S3, available as supplementary data at *Bioinformatics* online). *pileup-hi* was also validated with regression tests from the original *samtools mpileup* regression test suite, passing all tests across a variety of input arguments (e.g. -A, -B, -x, -aa, -f –rf, —ff, -r) and varying thread counts; this included working with SAM, BAM, and CRAM versions of the same input file, combining output generated from multiple input files, and using BAQ and depth limits (Table S4, available as supplementary data at *Bioinformatics* online).

Interestingly, *sambamba mpileup* yielded identical output hashes to 2/5 files (T. pallidum WGS and Rocoto pepper): despite using the same parameters across all tests, we observed single-coordinate differences between *sambamba* and *samtools*, where the former was lower in depth/base counts, presumably due to partial fetching of BAM regions designated to each thread.

### 3.3 Alternative pileup-hi histo output saves up to 99% of space occupied by standard pileup

Many downstream applications of the pileup algorithm, such as variant calling, do not always require nucleotide lists and quality scores; rather, they can rely on aggregate nucleotide counts at a given position. Because this format doesn’t grow linearly with alignment depth and omits base qualities, it can save significant space when applied to deep sequencing experiments where the extra information of the pileup is not required. *pileup-hi* supports this condensed output, named ‘histo’, generated directly from in-memory data. We compared the output size of the standard pileup and histo formats across all test datasets and observed up to a 99.8% reduction in file size (Fig. 1F, Table S4, available as supplementary data at *Bioinformatics* online). For example, the *T. pallidum* sample outputs reduced in size from 30 GB down to 49 MB, coinciding with their relatively short reference length (1.1 Mbp) and high depth (mean = 20 253). Human WGS samples yielded size reductions of 145–998 GB (58.2–89.9%), while the relatively low-depth (mean depth = 8.5) pepper genome dataset saw only a 4 GB reduction (2.8%).

## 4 Discussion

We present *pileup-hi*, a fast, scalable and customizable pileup engine for retrieving alignment-aware nucleotide information from the SAM/BAM file formats. *pileup-hi* generates binary-identical output to the established pileup software *samtools mpileup*, has a similarly small memory footprint (below 4 GB even for deep human sequencing) and is up to 13× as fast. *pileup-hi* also offers up to a 2.6× speedup compared to a parallel implementation of *samtools mpileup* at equivalent thread counts. When compared to *perbase*, a new and multi-threaded Rust pileup parser using the htslib pileup engine, *pileup-hi* is up to 8× as fast and uses up to 40× less memory.

Because reading a BAM file for reference-alignment differences is a universal need across experiments with highly variable aims (e.g. resistance mutation screening, variant calling, cancer detection), *pileup-hi* is built to be modular: users can create their own output formats to deliver the information they need directly, skipping the expensive, space-intensive generation of the pileup format if necessary. *pileup-hi* includes one such output format, ‘histo’, that returns a count of nucleotides and indels rather than a list, saving up to 99% of disk space: For experiments where per-query nucleotide and quality information is not necessary (e.g. some variant-calling and consensus generation workflows), ‘histo’ can avoid significant overhead from writing large amounts of data to disk. If this output proves too sparse for users, they can pipe *pileup-hi* output into compression algorithms to save space, or use the interface provided to develop their own formats.

While *pileup-hi’s* performance and modularity improvements offer users more agency and fewer computational bottlenecks in the processing of their data, limitations should be considered. *pileup-hi* offers only BAQ as a means for filtering poor-quality alignments, which may not be ideal for resolving all alignment artifacts. Stratifying input by read group is not yet supported. While we demonstrate significant performance gains with *pileup-hi*, speed improvements plateau with increasingly high thread counts: This suggests that the core pileup algorithm and multi-threading strategy should be revisited in the future for greater parallelism.

The decreasing cost of the sequencing run and the increasing adoption of high-accuracy, high-yield sequencers in time-sensitive contexts such as clinical testing will continue to exacerbate existing computational bottlenecks in bioinformatics pipelines: raw speed in single-threaded workloads, scalable and efficient multi-threading approaches to existing algorithms, and the circumvention of disk write and capacity constraints are increasingly critical areas for improvement. Future work will explore improving existing, fundamental bioinformatics algorithms to address the rising torrent of sequencing data.

## Supplementary Material

btag721_Supplementary_Data

## Data Availability

Data and scripts associated with this article can be accessed at Zenodo DOIs https://doi.org/10.5281/zenodo.19612806, https://doi.org/10.5281/zenodo.19613934, https://doi.org/10.5281/zenodo.19614468, https://doi.org/10.5281/zenodo.21480618, https://doi.org/10.5281/zenodo.21480649, https://doi.org/10.5281/zenodo.21480662, and GitHub repository https://github.com/greninger-lab/pileup-hi_paper. For regression tests and associated instructions, visit the tool’s main repository: https://github.com/greninger-lab/pileup-hi.

## References

[btag721-B1] Almogy G , Pratt M, Oberstrass F et al Cost-efficient whole genome-sequencing using novel mostly natural sequencing-by-synthesis chemistry and open fluidics platform. bioRxiv, 10.1101/2022.05.29.493900, 10 August 2022, preprint: not peer reviewed.

[btag721-B2] Beale MA , Marks M, Sahi SK et al Genomic epidemiology of syphilis reveals independent emergence of macrolide resistance across multiple circulating lineages. Nat Commun 2019;10:3255. 10.1038/s41467-019-11216-7. PMC6646400 31332179

[btag721-B3] BLAKE3 team. BLAKE3-Team/BLAKE3, Assembly. 10 Nov. 2019. 2026. https://github.com/BLAKE3-team/BLAKE3. (27 February 2026, date last accessed).

[btag721-B4] Cheng AP , Widman AJ, Arora A et al Whole genome error-corrected sequencing for sensitive circulating tumor DNA cancer monitoring. bioRxiv, 10.1101/2022.11.17.516904, 17 November 2022, preprint: not peer reviewed.

[btag721-B5] Danecek P , Bonfield JK, Liddle J et al Twelve years of SAMtools and BCFtools. Gigascience 2021;10:giab008. 10.1093/gigascience/giab008 PMC7931819 33590861

[btag721-B6] Eisfeldt J , Ameur A, Lenner F et al Towards routine long-read sequencing for rare disease: a national pilot study on chromosomal rearrangements. medRxiv, 10.1101/2023.12.15.23299892, 15 April 2024, preprint: not peer reviewed.

[btag721-B7] Höjer P , Alneberg J, Lundin P et al Whole genome sequencing with AVITI and NovaSeq X Plus reveals comparable performance with contextual biases. *NAR Genomics Bioinf* 2025;**8**:lqag053. 10.1093/nargab/lqag053 PMC13202175 42206012

[btag721-B8] Illumina. XLEAP-SBS chemistry enables Q40 and above data quality on NovaSeq X and NextSeq 1000/2000. 2024. https://www.illumina.com/content/illumina-marketing/amr/en_US/science/genomics-research/articles/data-quality-q-scores.html. (7 April 2026, date last accessed).

[btag721-B9] Illumina. NovaSeq X Specifications | Capacity for high-intensity genomics. 2026. https://www.illumina.com/systems/sequencing-platforms/novaseq-x-plus/specifications.html. (7 April 2026, date last accessed).

[btag721-B10] Ishida S , Sato Y, Chosa K et al CRISPR–Cas3-based editing for targeted deletions in a mouse model of transthyretin amyloidosis. Nat Biotechnol 2026:1–11. 10.1038/s41587-025-02949-6 41491255

[btag721-B11] Khanna A , Larson DE, Srivatsan SN et al Bam-readcount - rapid generation of basepair-resolution sequence metrics. J Open Source Softw 2022;7:3722. 10.21105/joss.03722 PMC13367885 42453900

[btag721-B12] Kim S , Scheffler K, Halpern AL et al Strelka2: fast and accurate calling of germline and somatic variants. Nat Methods 2018;15:591–4. 10.1038/s41592-018-0051-x 30013048

[btag721-B13] Li H , Handsaker B, Wysoker A et al The Sequence Alignment/Map format and SAMtools. Bioinformatics 2009;25:2078–9. 10.1093/bioinformatics/btp352 PMC2723002 19505943

[btag721-B14] Liu F , Zhao J, Sun H et al Genomes of cultivated and wild Capsicum species provide insights into pepper domestication and population differentiation. Nat Commun 2023;14:5487. 10.1038/s41467-023-41251-4 PMC10484947 37679363

[btag721-B15] McDaniel JH , Patel V, Olson ND et al Development and extensive sequencing of a broadly-consented genome in a bottle matched tumor-normal pair. Sci Data 2025;12:1195. 10.1038/s41597-025-05438-2 PMC12267650 40670386

[btag721-B16] nf-core/viralrecon. Assembly and intrahost/low-frequency variant calling for viral samples. n.d. https://github.com/nf-core/viralrecon/. (7 April 2026, date last accessed).

[btag721-B17] Picard toolkit. *Broad Institute, GitHub Repository*. Broad Institute. 2019. https://broadinstitute.github.io/picard/

[btag721-B18] Polonis K , Blommel JH, Hughes AEO et al Innovations in short-read sequencing technologies and their applications to clinical genomics. Clin Chem 2025;71:97–108. 10.1093/clinchem/hvae173 39749506

[btag721-B19] Poplin R , Chang PC, Alexander D et al A universal SNP and small-indel variant caller using deep neural networks. Nat Biotechnol 2018a;36:983–7. 10.1038/nbt.4235 30247488

[btag721-B20] Poplin R , Ruano-Rubio V, DePristo MA et al Scaling accurate genetic variant discovery to tens of thousands of samples. bioRxiv, 10.1101/201178, 24 July 2018b, preprint: not peer reviewed.

[btag721-B21] Santuari L , Kolpakov I, Blin A et al 2025; Evaluation of the Ultima Genomics UG 100^TM^ sequencing platform for clinical services. bioRxiv, 10.64898/2025.12.11.693628, 12 Dec 2025, preprint: not peer reviewed.

[btag721-B22] Stadick S. Perbase: a performant per-base sequencing metrics toolkit with accurate handling of complex alignments. JOSS 2026;11:9774. 10.21105/joss.09774

[btag721-B23] Tarasov A , Vilella AJ, Cuppen E et al Sambamba: fast processing of NGS alignment formats. Bioinformatics 2015;31:2032–4. 10.1093/bioinformatics/btv098 PMC4765878 25697820

