# Supplementary material for: Pileup-hi: an ultra-high-throughput, customizable alignment pileup program for large datasets
Source: Bioinformatics. 2026 Sep 28;42(10):btag721. doi: 10.1093/bioinformatics/btag721 (PMC13656176; doi:10.1093/bioinformatics/btag721)
Supplement: Supplementary file 1 [file btag721_supplementary_data.zip › supp_pileup-hi_2026Aug13_clean-ag.docx]

**Supplementary Methods**

**Datasets and pileup-hi software**

*pileup-hi* and its regression tests are available in source and binary form from <https://github.com/greninger-lab/pileup-hi>. Detailed dependency information and installation instructions are listed on the page’s README. All code, scripts, and references/BAMs used in this paper can be found at <https://doi.org/10.5281/zenodo.19612806>, <https://doi.org/10.5281/zenodo.19613934>, <https://doi.org/10.5281/zenodo.19614468>, <https://doi.org/10.5281/zenodo.21480618>, <https://doi.org/10.5281/zenodo.21480649>, and <https://doi.org/10.5281/zenodo.21480662>. Scripts and run instructions for data analysis can also be found at <https://github.com/greninger-lab/pileup-hi_paper>.

**Benchmarking and output comparison**

All analysis was performed on a 2020 iMac with 72GB of memory and an Intel Core i9-10910 CPU with 10 cores and 20 threads running MacOS Sequoia 15.7.4. Benchmarking was performed via the bench.py script, output binary comparison via the compare_output.py script, and output size comparison via the compare_size.py script.

For all analysis we used *samtools* v1.2.3 + *htslib* v1.2.3 (Li *et al.* 2009, Danecek *et al.* 2021), *perbase* 1.2.0 (Stadick 2026), *pileup-hi* v0.9.5, *b3sum* 1.8.3 (BLAKE3 team [2019] 2026), minimap2 v2.30-r1290-dirty (Li 2018), *sambamba* 1.0.1 (Tarasov *et al.* 2015), *bam-readcount 1.0.1* (Khanna *et al.* 2022)*,* and python 3.14.2.

**Dataset preparation**

Code for aligning FASTQ reads to reference genomes can be found in the *aln.*sh document (uploaded to the aforementioned repositories). Reads for the “pepper” dataset were downloaded from SRA accession SRR19895870 and aligned to Grif_1614 chromosomes 1-12 (CM061538.1-CM061549.1) with *minimap2* *-ax sr* and filtered for unmapped reads with *samtools view -F 2052*, sorted and indexed. Reads for the “human” samples were downloaded from SRA accessions SRR36374445, SRR30646149, and DRR793869. These reads were aligned to a multi-reference FASTA comprised of human chromosomes 1-23, M, X, and Y obtained from the hg38 analysis set hosted on <https://hgdownload.cse.ucsc.edu/goldenpath/hg38/bigZips/analysisSet/>. The *T. pallidum* sample was downloaded from SRA accession ERR2756169 and put through the tp-variants pipeline (<https://github.com/greninger-lab/tpallidum-variant-calling>) (Merkel 2014, Ewels P *et al.* 2016, Di Tommaso *et al.* 2017, Kurtzer, Sochat, and Bauer 2017, Veiga Leprevost da *et al.* 2017, Grüning *et al.* 2018, Ewels PA *et al.* 2020, Anaconda, n.d., Babraham Bioinformatics - FastQC A Quality Control tool for High Throughput Sequence Data, n.d.) to generate ERR2756169_merged.bam, which was then used for benchmarking.

**References**

Anaconda. Advance AI with Open Source. n.d. https://www.anaconda.com/ (27 Feb. 2026, date last accessed).

Babraham Bioinformatics - FastQC A Quality Control tool for High Throughput Sequence Data. n.d. https://www.bioinformatics.babraham.ac.uk/projects/fastqc/ (27 Feb. 2026, date last accessed).

BLAKE3 team. *BLAKE3-Team/BLAKE3*, Assembly. 10 Nov. 2019, 27 Feb. 2026. https://github.com/BLAKE3-team/BLAKE3 (27 Feb. 2026, date last accessed).

Danecek P, Bonfield JK, Liddle J *et al.* Twelve years of SAMtools and BCFtools. *Gigascience* 2021;**10**(2):giab008. https://doi.org/10.1093/gigascience/giab008.

Di Tommaso P, Chatzou M, Floden EW *et al.* Nextflow enables reproducible computational workflows. *Nat Biotechnol* 2017;**35**(4):316–9. https://doi.org/10.1038/nbt.3820.

Ewels P, Magnusson M, Lundin S *et al.* MultiQC: summarize analysis results for multiple tools and samples in a single report. *Bioinformatics* (Oxford, England) 2016;**32**(19):3047–8. https://doi.org/10.1093/bioinformatics/btw354.

Ewels PA, Peltzer A, Fillinger S *et al.* The nf-core framework for community-curated bioinformatics pipelines. *Nat Biotechnol* 2020;**38**(3):276–8. https://doi.org/10.1038/s41587-020-0439-x.

Grüning B, Dale R, Sjödin A *et al.* Bioconda: sustainable and comprehensive software distribution for the life sciences. *Nat Methods* 2018;**15**(7):475–6. https://doi.org/10.1038/s41592-018-0046-7.

Khanna A, Larson DE, Srivatsan SN *et al.* Bam-readcount - rapid generation of basepair-resolution sequence metrics. *Journal of Open Source Software* 2022;**7**(69):3722. https://doi.org/10.21105/joss.03722.

Kurtzer GM, Sochat V, Bauer MW. Singularity: Scientific containers for mobility of compute. *PLoS One* 2017;**12**(5):e0177459. https://doi.org/10.1371/journal.pone.0177459.

Li H. Minimap2: pairwise alignment for nucleotide sequences. *Bioinformatics* 2018;**34**(18):3094–100. https://doi.org/10.1093/bioinformatics/bty191.

Li H, Handsaker B, Wysoker A *et al.* The Sequence Alignment/Map format and SAMtools. *Bioinformatics* 2009;**25**(16):2078–9. https://doi.org/10.1093/bioinformatics/btp352.

Merkel D. Docker: lightweight Linux containers for consistent development and deployment. *Linux J* 2014;**2014**(239):2:2.

Stadick S. perbase: A performant per-base sequencing metrics toolkit with accurate handling of complex alignments. *Journal of Open Source Software* 2026;**11**(119):9774. https://doi.org/10.21105/joss.09774.

Tarasov A, Vilella AJ, Cuppen E *et al.* Sambamba: fast processing of NGS alignment formats. *Bioinformatics* 2015;**31**(12):2032–4. https://doi.org/10.1093/bioinformatics/btv098.

Veiga Leprevost F da, Grüning BA, Alves Aflitos S *et al.* BioContainers: an open-source and community-driven framework for software standardization. *Bioinformatics* (Oxford, England) 2017;**33**(16):2580–2. https://doi.org/10.1093/bioinformatics/btx192.
